# The Income Gradient in Mortality during the Covid-19 Crisis: Evidence from Belgium

**DOI:** 10.1007/s10888-021-09505-7

**Published:** 2021-08-26

**Authors:** André Decoster, Thomas Minten, Johannes Spinnewijn

**Affiliations:** 1 Department of Economics, Katholieke Universiteit Leuven, Belgium; 2 Department of Economics, London School of Economics, United Kingdom

**Keywords:** Inequality, Mortality, COVID-19

## Abstract

We use population-wide data from linked administrative registers to study the distributional pattern of mortality before and during the first wave of the Covid-19 pandemic in Belgium. Over the March-May 2020 study period, excess mortality is only found among those aged 65 and over. For this group, we find a significant negative income gradient in excess mortality, with excess deaths in the bottom income decile more than twice as high as in the top income decile for both men and women. However, given the high inequality in mortality in normal times, the income gradient in all-cause mortality is only marginally steeper during the peak of the health crisis when expressed in relative terms. Leveraging our individual-level data, we gauge the robustness of our results for other socioeconomic factors and decompose the role of individual vs. local effects. We provide direct evidence that geographic location effects on individual mortality are particularly strong during the first wave of the Covid-19 pandemic, channeling through the local number of Covid infections. This makes inference about the income gradient in excess mortality based on geographic variation misguided.

**Supplementary Information:**

The online version contains supplementary material available at 10.1007/s10888-021-09505-7.

## 1 Introduction

The Covid-19 pandemic affects everyone, but not everyone is affected equally. An important concern is that the burden of the Covid-19 crisis falls disproportionately on people with low income or socioeconomic status. A burgeoning literature studying the economic impact of the Covid-19 crisis and the associated policy measures on employment, earnings, and consumption (e.g., Adams-Prassl et al. 2020, Bachas et al. 2020, Chetty et al. 2020)

We thank Statbel, and especially Patrick Lusyne and Lien Tam Co for help with the data. Chloé de Meulenaer, Sebastian Ernst and Stijn Van Houtven provided excellent research assistance. We gratefully acknowledge funding by ERC (grant #716485) and by the Belgian Ministry of Social Security (2020-DGSTRAT-Studie Covivat).

✉ Johannes Spinnewijn
j.spinnewijn@lse.ac.uk

1 Department of Economics, Katholieke Universiteit Leuven, Belgium

2 Department of Economics, London School of Economics, United Kingdom documents substantial differences depending on socioeconomic status. In parallel, many research efforts have focused on the inequality of the health impact of the pandemic. While a rapidly growing literature suggests that socioeconomic factors are important determinants of Covid-19-related mortality (e.g., Chen et al. 2020, Drefahl et al. 2020, Jung et al. 2020 and Williamson et al. 2020), a strong income and/or socioeconomic gradient in health outcomes and in mortality in particular was present prior to the arrival of the coronavirus. Indeed, one seemingly perennial finding documented in many countries is that mortality rates are higher among individuals with lower socioeconomic status (e.g., Chetty et al. 2016, Mackenbach et al. 2019). An outstanding question is how the Covid-19 crisis has affected this relationship between income or socioeconomic status and mortality in particular. Lower income households may have been more exposed to the virus, for example because of their living or work conditions, but may also have medical conditions that put them more at risk when infected.

To answer this question we use population-wide data drawing from several administrative registers in Belgium. Belgium has been hit particularly hard by the first wave of the pandemic, noting the highest per capita death toll of any country by 30 May 2020. We use the mortality registers updated until June 2020 and linked to income registers as well as other demographic and socioeconomic information. This allows us to measure the income and socioeconomic gradient in mortality at the individual level, which we compare during the height of the Covid-19 health crisis - from March until May 2020 - with the corresponding months from 2015 to 2019.

A first advantage of our data on all-cause mortality is that we can perform a counterfactual analysis comparing mortality during and before the crisis. This allows us to provide evidence of the unequal burden of mortality due to the Covid-19 pandemic and relate it to the “usual” inequality in mortality in Belgium. A large number of papers, as shown in the left column of Table 1, have used Covid-19-related deaths counted by the health authorities, mostly finding stark differences in mortality across different socioeconomic groups. But, importantly, not knowing the counterfactual mortality, these studies cannot infer how the Covid-19 pandemic has affected inequality in mortality.

A second advantage of our data is that we can measure income and mortality at the individual level and therefore separate individual income-related factors from location effects. This is important because the correlation between mortality and household income may be driven by many factors, not in the least the location one lives in, and the importance of these factors may have changed during the pandemic. At the local level, there have been clear differences in the inflows of infected individuals and the propagation of infections. Moreover, healthcare capacity varies across localities, leading to differences in access to healthcare during the pandemic. These local factors may translate into differential mortality at different income positions. But we can also expect differences in exposure to infections by income or socio-economic status at the individual level (e.g., due to differences in employment, housing, social contacts, etc). In addition, individuals at different income levels have different co-morbidities and hence a different case fatality risk once infected.

The rows of Table 1, however, show that all but one paper analyze the relationship between mortality and socioeconomic status measured at the municipality or another location-specific level in various countries. While most studies find a negative association, some indicate a more ambivalent relationship.[1] An important limitation of studies that use

[1] Brandily et al. (2020), for instance, investigate excess mortality across municipalities in France, and find a negative income gradient, with excess mortality in the poorest municipalities twice as large as in other municipalities. In contrast, Jung et al. (2020) investigate the relationship between Covid-19 mortality and poverty aggregate measures, however, is that they do not measure the direct link between individuals' socioeconomic status and mortality. By looking at area-level measures, these effects may confound various local factors like access to and quality of care, exposure risk and also local policy responses.

As listed in Table 1, only a few studies have looked at excess mortality, but using aggregate data, and only one study has used individual data, but looking at Covid-19-related mortality. This notable exception is the study by Drefahl et al. (2020), finding a negative association between individual income from Swedish registries and Covid-19-related deaths. There is thus a gap in the literature studying the relationship between individual-level measures of socioeconomic status and excess mortality during the Covid-19 crisis. Our paper aims to fill this gap and provides three main sets of results:

First, when looking at the entire March-May 2020 period, we find only slight non-significant excess mortality for people under 65 in Belgium during the first wave of the Covid-19 crisis. While (EuroMOMO 2020) do find significant excess mortality for 45-64 year olds in Belgium during some weeks in the Covid-19 crisis, when we look at the entire March-May 2020 period, we do not find significant excess mortality for this age group. Our findings for the 45-64 age group in Belgium contrast with findings for this age group in other countries studied in EuroMOMO (2020). We also do not find a meaningful change in the income gradient of all-cause mortality for this demographic group compared to the baseline years. The ratio between mortality among the bottom income and the top income decile stayed around 5 for men and 4 for women. In light of the earlier evidence on the unequal incidence along the income distribution in this age group of both Covid-19-related mortality (e.g., Drefahl et al. 2020) and the underlying risk factors (e.g., Raifman and Raifman 2020, Wiemers et al. 2020), this may come as an unexpected result.

Second, our results show that the Covid-19 pandemic significantly affected the mortality of individuals aged 65 and over, and that excess mortality for this age group declines significantly with income. For example for men, we estimate 326 excess deaths out of 100,000 in the bottom income decile compared to 131 in the top income decile. Importantly, the income gradient in mortality is strongly negative in normal times too. As a result, expressed in relative terms, the income gradient in all-cause mortality is only marginally steeper during the peak of the health crisis. We compare different measures for judging the inequality in all-cause mortality that the Covid-19 pandemic brought to this subpopulation, but can only reject lower-than-normal mortality inequality during its peak. Overall, our results for this age group are confirmed when looking into other socioeconomic factors. We find strong educational gradients in excess mortality, as elderly who did not complete primary school experienced higher increases in mortality rates (30.47%) than elderly with higher education (21.91%). The increase in mortality has also been higher among Italian-, Turkish- and Polish-born residents than among Belgian-, German- and Dutch-born residents. We study individuals living in nursing homes separately, as excess mortality during the Covid-19 pandemic has been particularly high for this subgroup, but we do not find any income gradient in mortality before or during the Covid-19 crisis for them.

across US counties and find that poverty and mortality are positively related in areas of low population density. In areas of high population density, however, they find a U-shaped relationship. Knittel and Ozaltun (2020) also analyze the county-level relationship between Covid-19 mortality and poverty in the US but find no correlation. They even find a *positive* relationship between mortality and median home value. Desmet and Romain W. (forthcoming) find a positive correlation between Covid-19 cases or mortality and median household income in US counties in the first months of the pandemic, that has turned negative afterwards.

**Table 1** Findings on the Association Between Socioeconomic Status (SES) and Mortality during the Covid-19 Crisis

| | Covid-19 Mortality | Excess Mortality |
|---|---|---|
| Individual-level measure of SES | Drefahl et al. (2020)<br>*Negative association* - Sweden | |
| Aggregate measure of SES | Abedi et al. (2020)<br>*Negative association* - US<br>Ashraf (2020)<br>*Negative association* - World<br>Brown and Ravallion (2020)<br>*Negative association* - US<br>Chen and Krieger (2021)<br>*Negative association* - US<br>Desmet and Romain W. (forthcoming)<br>*Mixed results*[a] - US<br>Jung et al. (2020)<br>*Mostly negative association*[b] - US<br>Kim and Bostwick (2020)<br>*Negative association* - US<br>Knittel and Ozaltun (2020)<br>*No/positive association*[c] - US<br>Office for National Statistics (2020)<br>*Negative association*[d] - UK<br>Sá (2020)<br>*Mixed results*[e] - UK<br>Tubadji et al. (2020)<br>*Negative association* - UK<br>Williamson et al. (2020)<br>*Negative association* - UK | Brandily et al. (2020)<br>*Negative association* - France<br>Calderón-Larrañaga et al. (2020)<br>*Negative association* - Sweden<br>Chen et al. (2020)<br>*Negative association* - US |

This table classifies the existing applied work on the relationship between SES and Covid-19-induced mortality into four quadrants, depending on the measure of mortality and SES used. Noted under each reference are the observed relationship between SES and Covid-19-induced mortality, as well as the country, in which the study was conducted. For papers that did not find a clear association, we provide further details below

[a]Desmet and Wacziarg find that a higher level of Covid-19 mortality in a county was positively correlated with median household income in the first months after the onset of the pandemic, but the correlation turned negative afterwards. They also report the relationship with measures of poverty and educational attainment

[b]The authors find a U-shaped relationship between Covid-19 mortality and SES in counties with high population density and a negative relationship in counties with low population density

[c]Knittel and Ozaltun find no correlation between Covid-19 death rate and poverty rate but find a positive correlation between Covid-19 death rate and median home value

[d]The authors look at both Covid-19 mortality and all-cause mortality, but do not examine excess mortality

[e]Sá finds no simple correlation between deprivation and Covid-19 mortality. Regression results show Covid-19 mortality to be higher in more deprived areas, although the relationship disappears when controlling for self-reported health

Third, we try to separate the role of individual and local effects in determining the income gradient and to investigate whether their role has changed during the crisis. As mentioned, most prior work has been constrained by data availability and only considers differences in mortality by income aggregated at some local level. We find that our estimates of the income gradient using household income are robust to the inclusion of municipality fixed effects during the baseline years. This indicates that in the Belgian context, geographical differences in healthcare do not explain much of the pre-Covid income gradient in mortality. However, during the Covid-19 crisis, location becomes more important and explains about half of the increase in the income gradient at the household level. The relation between mortality and municipality income itself doubles during the crisis. This increase is much larger than measured at the individual level. Interestingly, this increase can be fully explained by differences in Covid-19 infections at the municipality level. Importantly, but not unexpectedly, inference relying on geographical variation about the individual socioeconomic factors of mortality during the pandemic would be misguided.

The paper proceeds as follows. Section 2 discusses the data and context. Section 3 present our main results, starting with the income gradients of all-cause and excess mortality and discussing the inequality implications, then studying other socioeconomic factors and the role of location effects. Section 5 concludes.

## 2 Data and setting

Our study focuses on Belgium, which has been faced with a high count of Covid-19-related deaths per capita. The introduction of the Covid-19 virus in Belgium has mostly been attributed to the return of ski tourists from Italy and Austria after the national holiday week from February 22 until March 1, 2020. In response to the quick surge of Covid-19 infections that followed, a nationwide lockdown was imposed from March 18. This was slowly phased out starting with the opening of garden stores and DIY stores on the 18th of April, followed by the staggered opening of selected sectors (May 4), retail stores (May 11), and cafes and restaurants (June 8). At the same time, there was a staggered loosening of the restrictions on the number of close social contacts citizens could maintain with individuals from other households, going from 2 (May 4), to 4 (May 11) and 10 (June 8). These policy measures during the first months of the Covid-19 crisis were set at the federal level with arguably limited variation at the local level.

To study mortality across the income distribution, we link administrative data on mortality from the national register with data on income from tax records. We also link this to data from other population-wide registers, including the 2011 census. Below, we discuss the different data sources, which have been linked and made available through the Belgian Statistical Institute (Statbel). We start by briefly sketching the Belgian health system against the background of a strong welfare state.

**Health system and inequality in Belgium** Most inequality and poverty statistics for Belgium stand in sharp contrast with the conclusions for many other countries that inequality, poverty, material deprivation and insecurity are on the rise. OECD (2018) reports a minor change in the Gini from 0.257 in 1983 to 0.264 in 2011, and even a slight decline since 2004.[2] The risk-of-poverty rate has also remained stable during the last decades. Although

[2]Based on survey data, Van Rie and Marx (2014) conclude that Belgian income inequality remained fairly stable between 1985 and the late 2000s. Decoster et al. (2017) also do not find evidence that those at the top a comprehensive explanation for these findings is still lacking, the strength of different components of the welfare state, such as labour market institutions, redistributive taxes and a high level of social protection financed by a high level of social insurance contributions, undoubtedly play their role. The Belgian healthcare system is another exponent of the strong welfare state with a combination of near universal coverage, regulated choice by both patients and healthcare providers, and no severe capacity constraints (hospital beds, doctors, nurses). Belgians enjoy a relatively high life expectancy and on average their self-reported health is high (5th place in the ranking of 28 EU-countries, see OECD/European Observatory on Health Systems and Policies (2017), Figure 4). But like in other developed countries, considerable socio-economic inequalities remain in health outcomes and behaviors, such as in self-reported health and in Covid-relevant unhealthy lifestyles such as smoking, alcohol use, diets and lack of physical activity. Despite the near universal coverage, Bouckaert et al. (2020) also point to an important socio-economic gradient in self-reported unmet care needs for financial reasons.

**Mortality in Belgium** Using the mortality records from the national register, Appendix Fig. A.1 shows the dramatic increase in daily deaths in March to May 2020 following the onset of the Covid-19 pandemic. To investigate the effect of the Covid-19 pandemic and associated policy responses on mortality, we consider its impact on all-cause mortality and define excess mortality as the difference in mortality between 2020 and the average mortality in the corresponding period from 2015 to 2019. Positive excess mortality in 2020 primarily occured from March 16 to May 27, with a record number of 314 excess deaths recorded on April 10. Another period of significant excess mortality occurred between August 8 and August 20, yet is ascribed to a heat wave that lasted from August 5 to August 17. We therefore take only the March-May period as the relevant period with which to compare mortality during the Covid-19 crisis in 2020 to the baseline years.[3] We note that total excess mortality in Belgium in this period is 8,195, which is close to the official number of Covid-19 deaths of 9,467 counted by Belgian health authorities.[4]

**Income** The income data originate from IPCAL, an administrative database that is drawn from personal income tax records. We use total net taxable income, which refers to income before tax, after social security contributions have been paid and costs deducted. It is a general definition of income, and includes labour income, unemployment benefits, sickness benefits and pensions.[5] Income data retrieved from tax declarations are contingent upon the tax legislation. Since capital income is subject to a liberating withholding tax, and of the income distribution in Belgium have benefited disproportionately from the economic growth since the nineties.

[3] Mortality was significantly higher than in the previous five years continuously between March 21 and May 21, between May 22 and May 25, and between August 8 and August 20.

[4] We do find a 13% discrepancy between excess mortality and the official death count (see also Molenberghs et al. 2020). Potential reasons for this discrepancy are the decrease in other-cause mortality in the study period, but also the over-counting of the Covid-19 death toll. Famously, all deaths with suspected involvement of Covid-19 were counted as Covid-19 deaths in Belgium. This has been actively portrayed as one of the reasons why the published death toll of Covid-19 in Belgium is one of the highest in the world.

[5] Pension income in Belgium is complex, and our data source based on taxable income captures annual pension income imperfectly. Pensions of the dominant 'first pillar' (the social security benefits) are a direct function of prior labor earnings and are mostly observed in the data. However, the treatment of the occupational pensions (the 'second pillar') and the personal private savings (the 'third pillar') is more problematic. Not only are these benefits only partly taxable in highly complex schedules, but tax payers can opt for the payment of this pension as a once-off lump sum amount. We find, nevertheless, that the correlation between some important benefits, such as child benefits, or the living wages (*leefloon*) are exempt from personal income tax, these income components are not included. We aggregate personal income over households to obtain household income. We do not aggregate income to the household level for individuals in nursing homes, as their household includes all other residents of the nursing home.[6]

**Demographic and Socioeconomic Variables** Most of the demographic information (age, country of birth, gender, municipality) originates from the national registries in Demobel. We also have an indicator for whether an individual is residing in a nursing home (*woonzorgcentra*) from Statbel. Economic sector and education level originate from the 2011 census. Municipality-specific information on per capita income and density comes from Statbel.

### 2.1 Excess mortality by age

Figure 1 contrasts mortality rates by age during the months March-May in 2020 and the corresponding period in 2015-2019. Panel A provides a clear visual picture of excess mortality across different ages, indicating how concentrated it has been among the elderly. Panel B zooms in on individuals aged 0 to 50 and shows that there was no significant excess mortality for people of those ages. Panel C zooms in on individuals aged 51-80 and shows that significant excess mortality only shows up for individuals aged 65+. These findings may seem surprising, as the Belgian health authorities (Sciensano) counted several hundred Covid-related deaths in the 45-64 age bracket. Note that there were a few weeks with significant excess mortality for 45-64 year olds (weeks 13-17, EuroMOMO (2020)), but these differences have been too small to lead to significant excess mortality over the March to May 2020 study period. This pattern has been documented before in Belgium (Molenberghs et al. (2020)) as well as in other countries (EuroMOMO (2020)). Only a few European countries, such as Spain and the UK, experienced large and significant excess mortality for people under 65 over a longer period. Clearly, returning to panel A, excess mortality is highest for individuals aged 80+.

Panel D considers nursing home residents separately and shows a substantial increase in mortality for nursing home residents aged 70+. Interestingly, this increase seems rather uniform for all ages above 70, which might be due to the selection of individuals less able to care for themselves into nursing homes, so that health status does not vary as much between older and younger nursing home residents compared to the general population. Our calculations suggest an especially heavy toll on nursing homes, as we estimate that in March to May 3.6% of all residents of nursing homes in Belgium died due to the Covid-19 pandemic.

Overall, we find important differences in excess mortality in Belgium across the age distribution during the Covid-19 crisis. Based on the different patterns in excess mortality, our results in Section 3 distinguish between individuals aged 40-64, individuals aged 65+ not living in nursing homes (or other collective households), and individuals aged 65+ who are living in nursing homes. Appendix Table A.1 provides summary statistics for the three samples. One way to aggregate the mortality effects throughout the age distribution is to calculate the *period* life expectancy, which is the life expectancy of an individual based our income measure when retired and earlier in life is quite strong, as evidenced by a high correlation of 0.63 between income decile at age 55 and income decile at age 65 for the same individual.

[6]The household indicators in our data come from the socioeconomic Demobel database. The income for individuals in nursing homes is dominated by pension income, as is the case for other 65+ year olds.

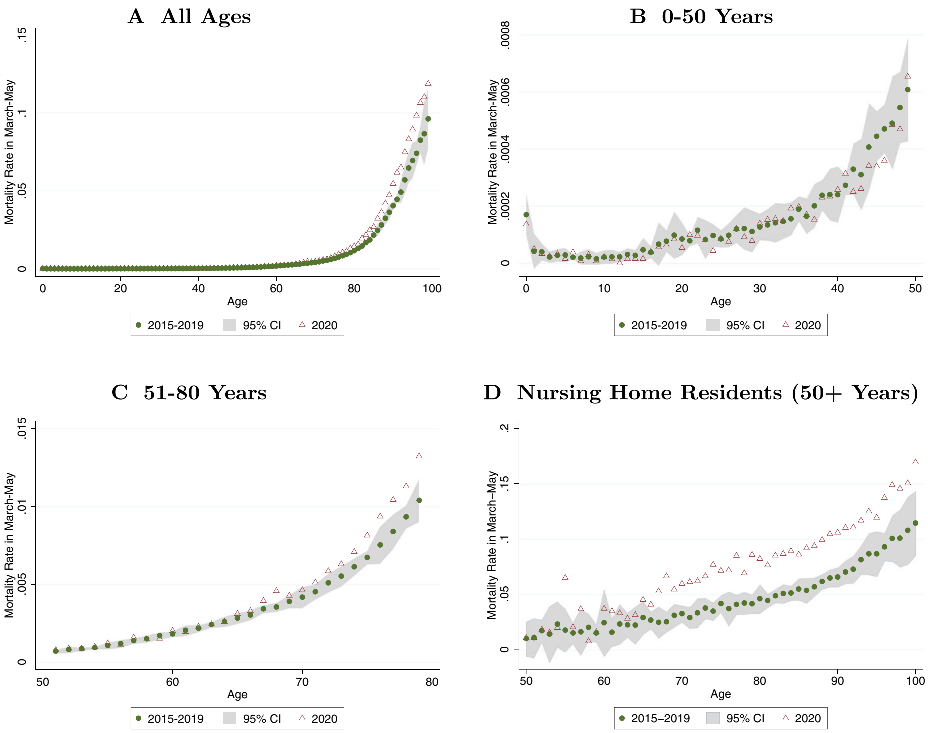

**Fig. 1** Mortality Rates in March-May By Age. These figures show the average mortality rate by age in March-May of 2015-2019, with a 95% confidence interval, and in March-May of 2020. Panels A-C show mortality rates for all Belgian inhabitants, excluding people living in collective households, or households with more than 10 individuals. Panel D shows mortality rates for nursing home residents according to the classification of Statbel on the age-specific mortality rates in a given period (e.g., Chetty et al. 2016). While the mortality rates increased the most for the elderly, changes in the mortality rates of the elderly have a smaller impact on life expectancy measures than changes among younger age groups. Based on the mortality rates in the baseline years, the period life expectancy in 2020 was 79.09 for men and 83.40 for women. Using the mortality rates between March-May of 2020 instead, the period life expectancy would be 1.87 years shorter for men, and 1.83 years for women.[7]

## 3 Income gradient of mortality rates

We now turn our analysis to the socioeconomic correlates of mortality and how their relationship changed during the Covid-19 crisis. Our main focus is on the income gradient of

[7]The period life expectancy for March-May 2020 is calculated in two steps. First, the 2015-2019 yearly mortality rates for each age-gender group are scaled with the P-score+1 obtained in March-May 2020, where the P-score is the estimated excess mortality divided by the baseline mortality within that group. Second, these scaled mortality rates are used to calculate life expectancy at birth for men and women separately.

mortality rates and in particular on the comparison of the income gradient during the Covid-19 crisis with that of the baseline years. Since income - and socioeconomic status more broadly - is central to equity considerations, a large literature has studied the importance of health inequality along this dimension. Importantly, income gradients by themselves do not allow one to draw any causal conclusions regarding the effect of income on health outcomes, either before or during the Covid-19 crisis. However, comparing the income gradients before and during the crisis sheds lights on how the crisis has affected health inequality along this dimension.

### 3.1 Income gradient before vs. during the Covid-19 crisis

To calculate the mortality-income gradient, we rank individuals based on their household income and calculate mortality rates for different income quantiles. In particular, for every year $t$, we rank individuals within their age-gender group based on their lagged household income in year $t-3$ and assign a decile based on these rankings. This means that the yearly deciles will be based on an individuals' lagged household income relative to all other individuals of the same gender and age in Belgium. We use a 3 year lag so that we observe lagged income for all years, including 2020, but by using lagged income we also reduce the potential response of income to health shocks (see Chetty et al. 2016) and in particular the response of income itself to the Covid-19 crisis.[8]

Figure 2 shows mortality rates for men and women of different age groups, both in the control years (2015-2019) and in 2020, across deciles. The slope of the income gradient, either using a linear or loglinear regression specification, corresponds to two commonly used inequality measures in the literature (see Mackenbach and Kunst 1997 and Moreno-Betancur et al. 2015): the $SII$ or Slope Index of Inequality and the $RII$ or Relative Index of Inequality respectively. Denoting mortality for decile $d$ by $m(d)$, $SII$ measures the difference $m(1) - m(10)$, and is often expressed in deaths per 100,000, whereas $RII$ is defined as the ratio $m(1)/m(10)$ or as the percentage change in mortality across the income scale. Appendix Table A.2 reports the slope estimates and the corresponding inequality indices for each of the income gradients.[9]

The top panels of Fig. 2 focus on individuals between 40-64 years old. The income gradient is already strong and negative in the baseline years. For men, the mortality rate is estimated to be 5.3 times higher in the bottom income decile than in the top income decile. The same holds for women, be it somewhat less outspoken with a corresponding $RII$ of 3.9. The negative income gradient in mortality rates is a persistent finding that underlies the substantial differences in life expectancy between low- and high income individuals (e.g., Chetty et al. 2016). Importantly, the figure shows that for this age group the mortality rates during the Covid-19 months are indistinguishable from those of the control months. While we documented above that there is no average excess mortality in this age group, the income gradients confirm that this is also true for individuals in different income groups.

The middle panels of Fig. 2 show a very different picture for the elderly. In the baseline years, the income gradient is again strongly negative. Compared to the younger age groups in panels A and B, the gradient is stronger when expressed in absolute terms, but smaller

[8]Calculating household income deciles based on one year only is appropriate, as we find that individuals' household income deciles remain relatively stable over time, a finding corroborated in Chetty et al. (2016). Importantly, we find that the high correlation between individuals' income deciles continues after retirement.

[9]In particular, with the estimated coefficient of the loglinear regression equal to $\beta$, we estimate the mortality ratio between the first and the tenth decile to be equal to $\frac{1}{(1+\beta)^9}$.

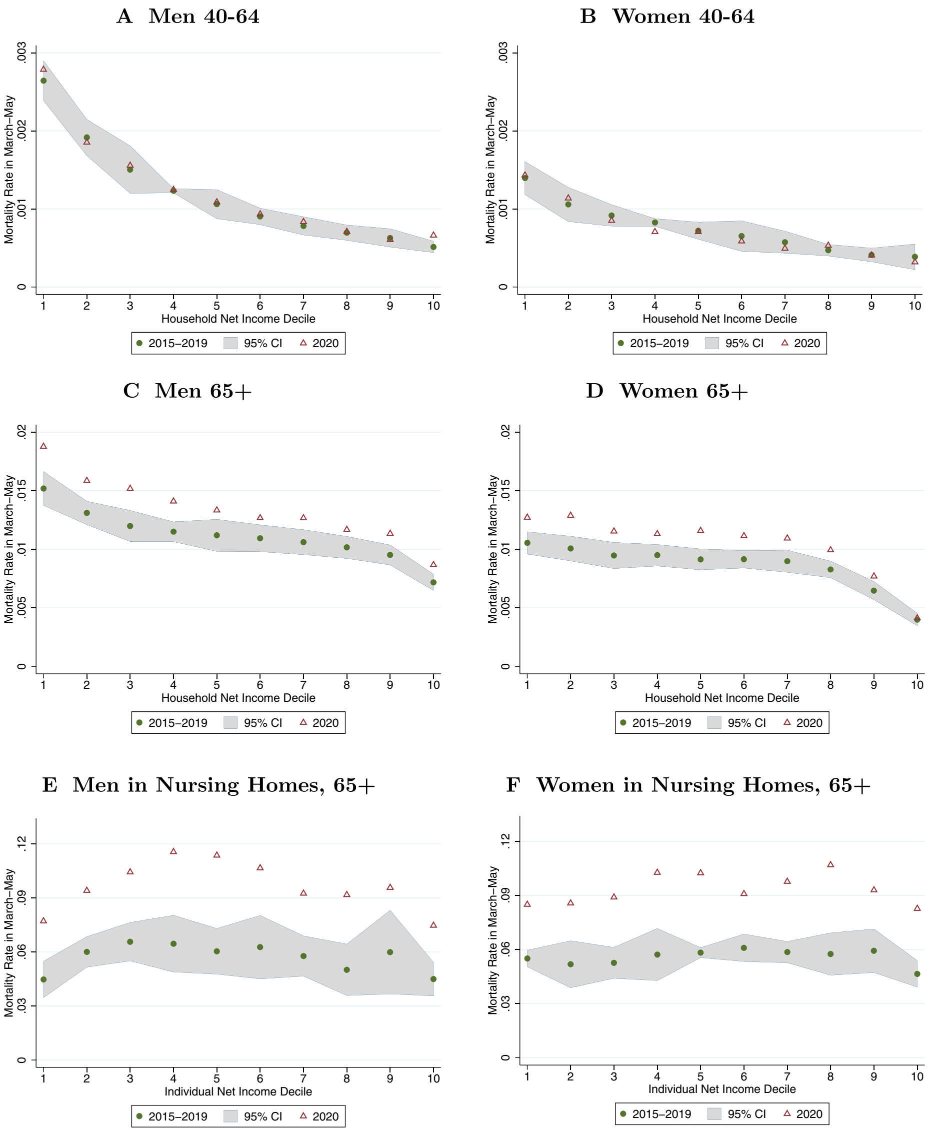

**Fig. 2** Mortality Rates in March-May By Gender/Age/Income. These figures show the average mortality rate by income decile in March-May of 2015-2019, with a 95% confidence interval, and in March-May of 2020. Panels A-D show mortality rates for all Belgian individuals, excluding people living in collective households or households with more than 10 individuals. Panels E and F show mortality rates for Belgian inhabitants aged 65 or older and living in nursing homes. These individuals are ranked based on their individual income within the corresponding age-gender group in the Belgian population, but to control for differential selection into nursing homes the results in Panels E and F are residualized on age when expressed in relative terms (see Table A.2 in Appendix). More importantly, the mortality rates jump significantly during the Covid-19 months and they do so in each of the income groups of this age group. The $SII$ increases substantially for men and women. For example, for men, the estimated difference in deaths of 596 per 100,000 individuals between the bottom and top income deciles during the baseline years increases to a difference of 791

deaths during the coronavirus period. However, expressed in relative terms, the increase in the income gradient has been more modest. The estimated $RII$ increases from 1.8 to 1.9 for men and from 2.1 to 2.3 for women.

Finally, the bottom panels show the mortality rates for individuals in nursing homes, who are excluded from the other panels. Interestingly, we do not find a clear income gradient in mortality rates for individuals in the baseline years. As is well known, mortality increased most starkly for this group, but it did so uniformly across income groups.

### 3.2 Distributional pattern of excess mortality

Several studies have analyzed the relationship between Covid-19-related mortality and socioeconomic status, arguing that the incidence of the pandemic falls disproportionately on low-income individuals. Our analysis of income gradients - before and during the Covid-19 crisis for different groups - nuances this view and provides a new perspective. We already noted above that different pictures emerge when presenting the gradient by means of absolute ($SII$) or relative ($RII$) differences across the income scale. Both measures correspond to a different ‘inequality equivalence’ when looking at changes, the $SII$ being invariant to equal absolute changes in mortality rates due to Covid-19, whereas the $RII$ is invariant to equal percentage changes across the income scale. The ‘choice’ of presenting excess mortality as an absolute difference or as a relative change between the baseline years and the Covid-19-period then boils down to the choice of an absolute or relative perspective for the income gradient.

The top panels of Fig. 3 show excess mortality - expressed in absolute terms - for each household income decile in the male and female populations older than 65. Consistent with the earlier observation that the $SII$ increased during the Covid-19 crisis, excess mortality, measured in absolute differences, is decreasing with income. The differences are substantial. Using the estimated linear income gradients in Appendix Table A.2, the estimated excess mortality is 326 out of 100,000 in the bottom decile vs. 131 in the top decile for men. The corresponding numbers are 269 vs. 96 for women.[10] This corroborates the argument that the mortality incidence of the Covid-19 crisis falls disproportionately on lower income households. The nuance is that the difference in excess mortality by income is entirely driven by the elderly. In the younger age group the negative income gradient in all-cause mortality has basically remained the same, while in the group of nursing home residents there has been no meaningful relationship between income and mortality, neither before nor during the Covid-19 crisis.

The bottom panels of Fig. 3 show excess mortality relative to baseline mortality - commonly referred to as P-scores (see Aron and Muellbauer 2020) - for each income decile within the same subgroups. The relationship between the relative mortality increase and income is less precise and less pronounced overall. This corresponds to the small and insignificant change in the $RII$ for both men and women, providing a new perspective on how much inequality has increased due to the Covid-19 crisis.

In principle it should not come as a surprise that choosing to use relative measures like the $RII$ or absolute measures like the $SII$, can lead to different conclusions. Whereas many authors conclude that the best way out of this uncomfortable choice of measure is to present several of them - illustrated by the numerous other measures described in Mackenbach and

[10]The difference in observed excess mortality is even larger, especially for women, as the observed excess mortality in the top decile is an outlier.

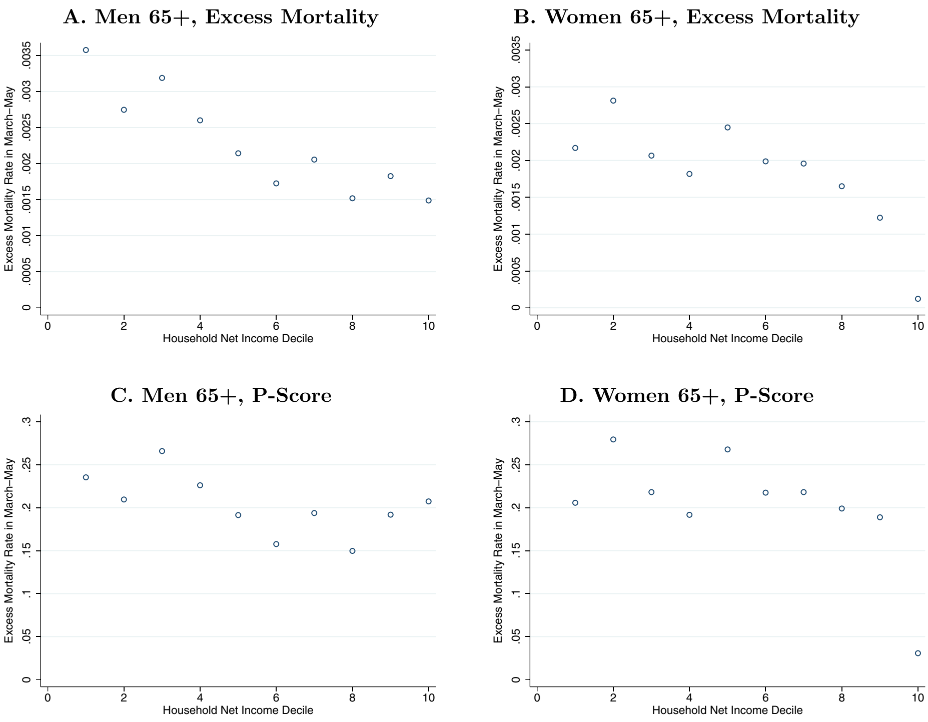

**Fig. 3** Income Gradient in Absolute and Relative Excess Mortality March-May 2020. Panels A-B plot the excess mortality rate by income decile in March-May 2020 for individuals aged 65 or older, excluding people living in collective households, or households with more than 10 individuals. Panels C-D show the excess mortality fraction (P-score) for the same groups of individuals, where the P-Score is defined as excess mortality in 2020 divided by average mortality in 2015-2019 within the associated group

Kunst (1997) - others point to the inescapable need to depart from the purely descriptive stance. They plead in favor of making the implicit value judgements in the chosen inequality measure explicit by following a more axiomatic route, inspired by the development in inequality or poverty measurement in the economic discipline.[11] Especially in the health economics context, this more axiomatic approach has been fruitful in unveiling the impact of using bounded variables (like mortality, which is bounded between 0 and 1), or the attractiveness of specific axioms, like the ‘mirror axiom’. The latter imposes that, whether one chooses to measure inequality in terms of an ‘attainment’ (e.g. ‘surviving’), or in terms of ‘shortfall’ (e.g. ‘dying’), one should obtain the same inequality ordering in distributional comparisons.[12] When following the index proposed by Erreygers (2009), satisfying the

[11]This is most markedly pronounced in the title of the paper by Kjellsson et al. (2015) ‘Lies, Damned Lies, and Health Inequality Measurements. Understanding the Value Judgements’. The descriptive nature of measures like $SII$ or $RII$ on the contrary, is revealed by labelling the estimated coefficients of the underlying regressions as the *least false parameter* (Moreno-Betancur et al. (2015) p.519), emphasising that these parameters not necessarily correspond to an estimate of a “true” model underlying the data.

[12]As shown by Erreygers (2009) and Erreygers and Van Ourti (2011), imposing the mirror axiom drastically reduces the choice of inequality measures to measures which are ‘absolute’ instead of ‘relative’, i.e. inequality is unaffected by equal additions or subtractions of the outcome variable across the income scale. The fact that one cannot satisfy scale invariance, when imposing the mirror principle is easily seen from the fact that mirror axiom, we again conclude that inequality has increased during the Covid-19 months (see Appendix Table A.2).

Besides the different normative perspectives, the obvious reason why the choice of measure matters so much empirically is the simple fact that mortality rates are so unequal during the baseline years. Framed differently: due to the strong baseline income gradient of mortality, the impact of the Covid-19 crisis on inequality is less clear cut. While it has not decreased by either of our measures, how much it has increased critically depends on the measurement of inequality.

### 3.3 Other socioeconomic determinants

An important strand of the literature on socioeconomic differences in health points to education as the go-to indicator of socioeconomic status. The reason for this is both pragmatic and fundamental. Education is often known in survey data, and as education is obtained early in life, it is a arguably less endogenous to health than income as a socioeconomic indicator. Panel A of Fig. 4 clearly shows how, for the elderly, the negative educational gradient in mortality becomes stronger during the Covid-19 crisis and the change is more pronounced than for the income gradient. Indeed, we find a negative educational gradient in excess mortality during the Covid-19 pandemic, both when expressed in absolute and relative terms. The mortality rate was 30.47% higher in March-May 2020 compared to the baseline years for elderly who did not complete primary school, while for elderly who completed higher education the increase was smaller at 21.91%. For individuals under 65, just like for the income gradient, the relationship between education and mortality remains largely unchanged during the Covid-19 pandemic, as shown in Appendix Fig. A.2.

We briefly consider two other socioeconomic factors in Panels B and C of Fig. 4:

First, several authors have documented the large burden of the pandemic on minorities in the US and UK (Bertocchi and Dimico (2020), Gross et al. (2020), McLaren (2020), Chowkwanyun and Reed (2020), Price-Haywood et al. (2020) and Chen and Krieger (2021)). While we do not observe race in our data, we do observe country of birth. Panel B of Fig. 4 shows the relative increases in mortality (P-values) for Belgian residents aged 65+ by country of birth for the 9 most represented countries as country of birth among the elderly in Belgium. The mortality increases among Belgian residents born in Italy (42.77%), Turkey (41.91%) and Poland (38.80%) are larger than among those born in Belgium (25.39%), Germany (23.21%) and Netherlands (6.80%).[13] We also investigate how much of this association is mediated by income. Appendix Fig. A.3 shows coefficients in a regression of excess mortality on country of birth after residualizing on income deciles. In Panel A, we find that the high excess mortality of people of Italian ethnicity is fully explained by differences in income. However, for individuals born in Morocco, Poland and Turkey the excess mortality is high, even conditional on income. That is, the estimated excess mortality is 0.33%, 0.16% and 0.13% higher for individuals born in these respective countries, above and beyond the potential differences in excess mortality explained by their income.

a distributional change which keeps the ratio's $m(i)/m(j)$ constant cannot simultaneously keep the ratio $(1-m(i))/(1-m(j))$ constant, where we use the example of mortality rates bounded between 0 and 1.

[13]When looking at excess mortality fractions for individuals aged 40-64 by country of birth in Appendix Fig. A.2, we generally find few groups with significantly positive excess mortality. One exception is the high and significant P-score of 52.79% for 40-64 aged Congolese-born individuals.

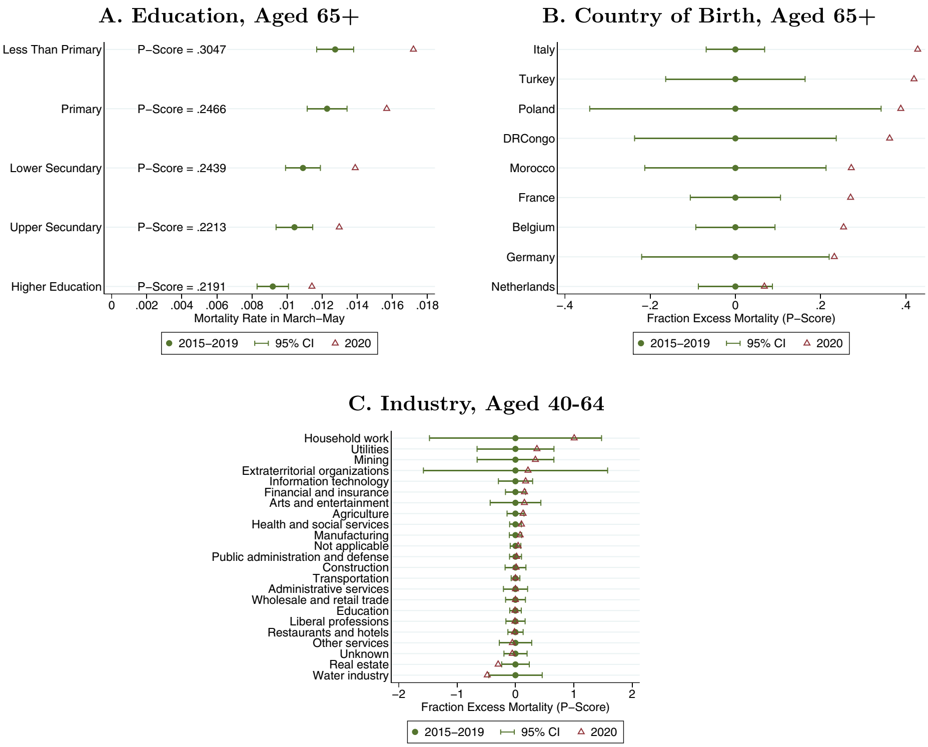

**Fig. 4** Excess Mortality by Education, Country of Birth and Industry. Panel A shows mortality rates (with 95% confidence intervals) in March-May 2015-2019 and March-May 2020 by educational level for individuals aged 65 and older. Excess mortality in percentages (P-Score) is also indicated on the figure. Panel B shows excess mortality fractions in March-May 2020 and 95% confidence intervals for 2015-2019 by country of birth for individuals aged 65 and older. Panel C shows excess mortality fractions in March-May 2020 and 95% confidence intervals for 2015-2019 by industry for individuals aged 40-64. Samples in all panels exclude individuals living in collective households, or households with more than 10 individuals. Average mortality rates (also used in the computation of the P-score) are the weighted average of mortality rates by age, where population-based weights are taken for each age. Such a calculation makes sure that there is no influence of age-related composition differences between origins on the plotted mortality rate differences or P-scores

Second, while we do not observe the occupation of workers, we do observe the industry they work in. Focusing on individuals between 40-64 years old, Panel C of Fig. 4 shows substantial dispersion in the relative increases in mortality across industries, but for none of the industries is the difference between the mortality rate during the Covid-19 crisis and the years before highly significant. This is not too surprising given the lack of significant excess mortality in that age group as a whole. Interestingly, the only sector where we do find marginally significant positive excess mortality is the health and social services sector (10.06%), where workers have arguably been more exposed to the virus. This potential explanation seems to be confirmed when we control for income (see Panel B Appendix Fig. A.3). Conditional on income, the association with excess mortality is highest for individuals working in utilities (2.1%) and in health and social services (1.1%), but lowest in industries that have been shut down during the lockdown, like real estate (-1.8%) and restaurants and hotels (-2.0%).

## 4 Individual vs. local effects

Our results so far show that an individual's mortality is highly correlated with his or her household income and that this correlation increased further in the first months of the Covid-19 crisis. An individual's income is, however, related to many other factors, in particular the location that he or she lives in. The pandemic has struck differently across locations with differences in the inflow, propagation and thus exposure to infections, but also with potential differences in access to hospitals and in response to the outbreak of the pandemic.

Most research studying the relationship between income or other socioeconomic factors and mortality during the Covid-19 crisis has been limited by data availability and needed to rely on aggregate measurements at different geographic levels (see Table 1). While the geographic inequality in the incidence of Covid-19 and how this correlates with income at the local level is important by itself, one should be cautious when drawing any inference about the role of individual socioeconomic determinants based on geographic variation. This would only be valid in the absence of local factors and geographic sorting on income. We illustrate this in Table 2, which reports the estimates from a regression of mortality over the March-May period on log income, allowing the relation to differ in the Covid-19 year 2020. Columns (1) and (7) compare the estimates when running this regressions at the individual vs. municipality level. Regressing individual mortality on log household income in column (1), we confirm the negative gradient we found before and how this negative gradient becomes significantly stronger in 2020. During the baseline years, the relationship between mortality and income is similar when measured at the individual level and the municipality level. However, this negative effect is more pronounced during the Covid-19 crisis when using municipality income than when using household income. In the former case, it almost doubles, while in the latter case, it increases by less than half. Hence, we would drastically overestimate the importance of socioeconomic factors at the individual level for excess mortality when using income measured at the municipality level.[14]

The individual income and mortality data allow us to go further and to separate the relationship between mortality and individuals' income from where individuals live and study how the role of individual vs. local factors changed during the crisis.[15] Column (2) in Table 2 repeats the regression of individual mortality on log household income, but includes age fixed effects in line with our graphical results shown before. This reduces the estimated gradient substantially, but simplifies its interpretation as it no longer captures the strong correlation between age and both income and mortality. In column (3) we add municipality times year fixed effects. Controlling for local factors, the estimates of the income effect at the household level remain very similar during the baseline years, but the increase in the income gradient during the crisis decreases and loses significance (p-value = 0.10).

[14] Appendix Table A.4 repeats the same analysis for the group of individuals aged 40-64. While there are no differences in excess mortality depending on household income, the effect of municipality income during the crisis is marginally significant (p-value = 0.052).

[15] Our decomposition exercise also relates to the separation of selection vs. place effects in explaining the geographic inequality in mortality (Finkelstein et al. (2019)).

**Table 2** Household Income Gradient vs. Municipality Income Gradient

| | Dependent Variable: | | | | | | |
|---|---|---|---|---|---|---|---|
| | Indiv. Mortality in March-May (0/1) | | | | | | Municip. Mortality Rate in March-May |
| | (1) | (2) | (3) | (4) | (5) | (6) | (7) |
| Log Household Income | –0.00419*** | –0.00101*** | –0.00097*** | –0.00092*** | –0.00094*** | –0.00094*** | |
| | (0.00006) | (0.00006) | (0.00006) | (0.00006) | (0.00006) | (0.00006) | |
| Year 2020 X Log Household Income | –0.00173*** | –0.00041*** | –0.00025 | –0.00019 | –0.00021 | –0.00023 | |
| | (0.00015) | (0.00015) | (0.00015) | (0.00015) | (0.00015) | (0.00015) | |
| Log Per Capita Municipality Income | | | | –0.00355*** | –0.00406*** | –0.00397*** | –0.00419*** |
| | | | | (0.00020) | (0.00030) | (0.00031) | (0.00027) |
| Year 2020 X Log Per Capita Municipality Income | | | | –0.00446*** | –0.00221*** | –0.00006 | –0.00395*** |
| | | | | (0.00054) | (0.00078) | (0.00081) | (0.00082) |
| Constant | 0.05270*** | 0.02124*** | 0.02055*** | 0.06243*** | 0.07031*** | 0.06580*** | 0.05099*** |
| | (0.00058) | (0.00054) | (0.00055) | (0.00187) | (0.00266) | (0.00276) | (0.00266) |
| Age-Time FE | NO | YES | YES | YES | YES | YES | NO |
| Municipality-Time FE | NO | NO | YES | NO | NO | NO | NO |
| Municipality Controls | NO | NO | NO | NO | YES | YES | NO |
| Number of Cases Control | NO | NO | NO | NO | NO | YES | NO |
| Observations | 12,156,397 | 12,156,396 | 11,619,380 | 11,613,489 | 11,613,489 | 11,608,535 | 3,372 |
| Adjusted R-squared | 0.00069 | 0.01202 | 0.01219 | 0.01207 | 0.01210 | 0.01211 | 0.24614 |

The invariance of the estimate in the baseline years when using only within-municipality variation indicates that the income gradient of mortality in Belgium is not driven by location effects. That is, the income gradient seems to reflect a relation between mortality and income itself rather than the effects of where individuals with different income live (e.g., accessibility and quality of healthcare). However, this is different during the pandemic. While imprecisely estimated, the smaller interaction terms in columns (2) and (3) suggest that location effects explain about half of the stronger relation between mortality and household income during the Covid crisis. This indicates that location effects have been important during the crisis indeed, but they cannot fully explain the stronger income gradient in mortality either.[16]

We can shed further light on the mechanisms underlying the stronger location effects. Instead of adding municipality fixed effects, column (4) adds average income at the municipality level, allowing again its relation with mortality to differ in 2020. Controlling for household income, individual mortality is higher in municipalities with lower average income and this municipality effect more than doubles during the pandemic. Column (5) in Table 2 shows how the municipality income effect during the pandemic is reduced when one controls for other demographic controls at the municipality levels, including the population density, share of elderly, share of elderly living in single households and share of immigrants. Interestingly, the extra effect of municipality income in 2020 disappears when we explicitly control for the number of Covid-19 infections in column (6), suggesting that location effects are important for infections, but not necessarily for case-fatality rates. The relationship between mortality and individual income, however, is robust to the specific controls for local factors. This again illustrates that any inference about individual relationships from analysis at a geographical level is difficult, but particularly so during a pandemic which plays out at the local level.

## 5 Discussion

This paper relates high-quality individual data on mortality to socioeconomic factors and contributes to a better understanding of the impact of the pandemic on the socioeconomic gradient of mortality. We showed that there exists a significant and negative income gradient in excess mortality during the Covid-19-period in Belgium for the elderly. However, this - strongly negative - gradient is comparable to the gradient in all-cause mortality in non-pandemic times. The Covid-19 crisis might stall the trend of narrowing absolute (but not relative) mortality inequality, as documented recently for European countries in Mackenbach et al. (2019).

The reasons for potential socioeconomic differences and thus a socioeconomic gradient in the incidence and mortality of Covid-19 are heavily debated. Despite the higher likelihood of high-income individuals to import the virus due to international travel, as shown in Pluemper and Neumayer (2020), several papers hint at higher transmission rates among individuals with low socioeconomic status once the illness is widespread within a country (e.g., Desmet and Romain W. (forthcoming)). Brandily et al. (2020) mention poor housing conditions and higher occupational exposure as the most likely mechanisms causing the higher burden for the poor in France, while McLaren (2020) stresses the importance of

[16]Note that when regressing mortality on income quartiles instead, again allowing for an interaction with a year 2020 dummy, the estimated interaction is also reduced when including municipality-time fixed effects, but the reduction is smaller and the interaction terms remains significant, as shown in Appendix Table A.3.

higher transit exposure among the less well off. Papageorge et al. (2021) argue that individuals of lower socioeconomic status typically have less flexible work arrangements and a lack of outside space at home, which in turn are correlated with less protection against a pandemic.

Due to the specific data-availability in the Belgian case, our current analysis faces important limitations. First, by using mortality data, we cannot separate the income gradient in infection (e.g., due to differences in employment or social contacts) from the one in case fatality risk (e.g., due to an income gradient in Covid-19 mortality risk factors). Clearly, linking the available data on hospitalizations, prior health diagnoses and test results would allow important progress to be made. Second, by considering mortality, we potentially miss out on important differences in morbidity, physical health, and mental well-being. Again, linking the available data from health records or surveys would allow researchers to provide a more comprehensive picture of the unequal consequences of the ongoing crisis.

**Supplementary Information** The online version contains supplementary material available at https://doi.org/10.1007/s10888-021-09505-7.

**Open Access** This article is licensed under a Creative Commons Attribution 4.0 International License, which permits use, sharing, adaptation, distribution and reproduction in any medium or format, as long as you give appropriate credit to the original author(s) and the source, provide a link to the Creative Commons licence, and indicate if changes were made. The images or other third party material in this article are included in the article's Creative Commons licence, unless indicated otherwise in a credit line to the material. If material is not included in the article's Creative Commons licence and your intended use is not permitted by statutory regulation or exceeds the permitted use, you will need to obtain permission directly from the copyright holder. To view a copy of this licence, visit http://creativecommons.org/licenses/by/4.0/.

## Electronic supplementary material

Below is the link to the electronic supplementary material.

(PDF 743 KB)
