# Supplementary material for: The Income Gradient in Mortality during the Covid-19 Crisis: Evidence from Belgium
Source: J Econ Inequal. 2021 Aug 26;19(3):551–70. doi: 10.1007/s10888-021-09505-7 (PMC8390079; doi:10.1007/s10888-021-09505-7)
Supplement: Supplementary file 1 — (PDF 743 KB) [file 10888_2021_9505_MOESM1_ESM.pdf]

ACCEPTED MANUSCRIPT

A Appendix Tables and Figures

Figure A.1: Covid-related Spike in Deaths in March-May 2020

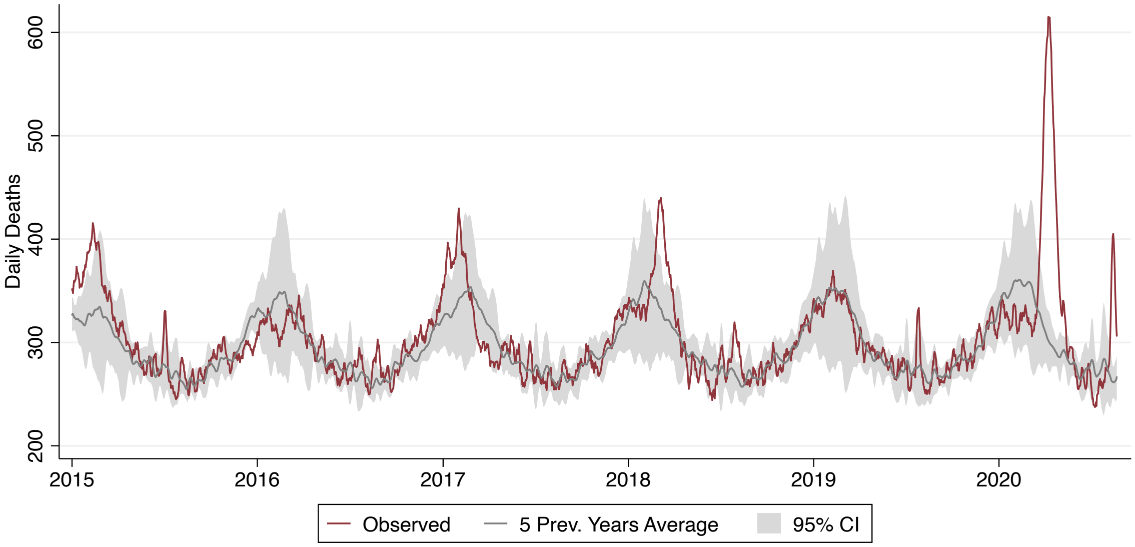

Notes: This plot shows the daily 7-day moving averages of the number of deaths recorded in Belgium. Also plotted is the average daily 7-day moving average of mortality in the 5 previous years, together with 95% confidence intervals.

Accepted manuscript

ACCEPTED MANUSCRIPT

Table A.1: Summary Statistics

	40-64	65+	Nursing Home Residents, 65+
	Mean	Mean	Mean
**Demographics**			
Male	49.97%	44.77%	24.97%
Age	52	75	86
Died in March-May 2020	0.10%	1.19%	9.64%
**Education Level**			
Missing	11.60%	8.01%	13.44%
Less Than Primary	1.52%	5.23%	7.60%
Primary	6.09%	20.45%	33.90%
Lower Secundary	16.95%	26.80%	23.98%
Upper Secundary	33.15%	20.79%	12.75%
Higher Education	30.69%	18.72%	8.34%
**Household Income**			
Mean	48,409	34,487	
p10	14,710	15,480	
Median	46,420	29,700	
p90	86,730	61,850	
**Personal Income**			
Mean	26,389	19,495	18,653
p10	10,440	8,380	12,870
Median	24,090	17,490	17,040
p90	50,000 (capped)	33,420	26,910
**Municipality**			
Per Capita Income	18,501	18,732	18,477
Number of Residents	66,343	64,415	72,120
Observations	3,740,619	2,130,114	100,829

Notes: This table shows summary statistics for three subsamples of Belgian citizens in 2020. Household and personal income are measured in 2017. Nursing home residents' household income is not included as in our data residents within one nursing home are counted as belonging to the same household. Municipality per capita income and number of residents are measured in 2017.

Accepted manuscript

ACCEPTED MANUSCRIPT

Table A.2: Regression and Inequality Estimates

	Aged 40-64		Aged 65+		Aged 65+ in Nursing Homes	
**A. Slope Estimates**	Men	Women	Men	Women	Men	Women
Linear Regression						
2015-2019	-0.00021	-0.00010	-0.00066	-0.00055	0.00152	-0.00084
	(0.00003)	(0.00001)	(0.00008)	(0.00011)	(0.00106)	(0.00041)
2020	-0.00020	-0.00011	-0.00088	-0.00075	0.00286	-0.00015
	(0.00004)	(0.00002)	(0.00009)	(0.00016)	(0.00202)	(0.00067)
Log-linear Regression						
2015-2019	-0.169	-0.139	-0.061	-0.077	0.030	-0.015
	(0.01)	(0.006)	(0.008)	(0.020)	(0.020)	(0.007)
2020	-0.158	-0.152	-0.067	-0.089	0.031	-0.002
	(0.015)	(0.012)	(0.007)	(0.025)	(0.020)	(0.007)
**B. Inequality Measures**						
Slope Index of Inequality (SII)						
2015-2019	185	93	596	499	-1368	758
2020	184	100	791	672	-2572	131
Relative Index of Inequality (RII)						
2015-2019	5.30	3.86	1.76	2.05	0.76	1.15
2020	4.71	4.43	1.86	2.31	0.76	1.02
Erreygers-index						
2015-2019	0.0014	0.0007	0.0044	0.0037	-0.0100	0.0056
2020	0.0013	0.0007	0.0058	0.0049	-0.0189	0.0010

Notes: This table provides information on the distributional pattern of mortality in 2015-2019 and in 2020. Panel (A) provides slope estimates and associated standard errors from a linear and log-linear regression of mortality rates on income deciles for both periods separately. Panel (B) shows several measures to evaluate the inequality in mortality in both periods. The calculation of SII - expressed per 100,000 - and RII are based on the estimated slopes in Panel (A).

Accepted manuscript

ACCEPTED MANUSCRIPT

TABLE A.3: INCOME GRADIENT WITHIN MUNICIPALITIES

	Dependent Variable:		
	Mortality in March-May (0/1)		
	(1)	(2)	(3)
Income Q2	-0.0015***	-0.0014***	-0.0014***
	(0.0001)	(0.0001)	(0.0001)
Income Q3	-0.0020***	-0.0020***	-0.0019***
	(0.0001)	(0.0001)	(0.0001)
Income Q3	-0.0044***	-0.0044***	-0.0043***
	(0.0001)	(0.0001)	(0.0001)
Year 2020 X Income Q2	-0.0004	-0.0002	-0.0002
	(0.0002)	(0.0002)	(0.0002)
Year 2020 X Income Q3	-0.0009***	-0.0007***	-0.0007***
	(0.0002)	(0.0002)	(0.0002)
Year 2020 X Income Q4	-0.0014***	-0.0012***	-0.0011***
	(0.0002)	(0.0002)	(0.0002)
Constant	0.0117***	0.0121***	0.0464***
	(0.0001)	(0.0001)	(0.0020)
Municipality-Time FE	NO	YES	NO
Log Municipality Income Control	NO	NO	YES
Observations	12,156,397	11,619,381	11,613,490
Adjusted R-squared	0.0003	0.0005	0.0004

Robust standard errors in parentheses

*** p<0.01, ** p<0.05, * p<0.1

Notes: This table regresses mortality in March-May on a year 2020 dummy and on household income quartile dummies, as well as their interactions for individuals aged 65 or older, excluding people living in collective households, or households with more than 10 individuals. Only observations from years 2015-2020 are included. Column (2) adds fixed effects for every Belgian municipality and their interactions with year 2020. Column (3) controls for the log per capita income in each municipality, as well as their interactions with year 2020.

Accepted manuscript

ACCEPTED MANUSCRIPT

Table A.4: Household Income Gradient vs. Municipality Income Gradient, Ages 40-64

	Dependent Variable:						
	Indiv. Mortality in March-May (0/1)						Municip. Mortality Rate in March-May
	(1)	(2)	(3)	(4)	(5)	(6)	(7)
Log Household Income	-0.00059***	-0.00056***	-0.00054***	-0.00054***	-0.00054***	-0.00054***	
	(0.00001)	(0.00001)	(0.00001)	(0.00001)	(0.00001)	(0.00001)	
Year 2020 X Log Household Income	-0.00001	-0.00002	-0.00002	-0.00001	-0.00000	-0.00000	
	(0.00003)	(0.00003)	(0.00003)	(0.00003)	(0.00003)	(0.00003)	
Log Per Capita Municipality Income				-0.00035***	-0.00063***	-0.00068***	-0.00087***
				(0.00005)	(0.00007)	(0.00007)	(0.00008)
Year 2020 X Log Per Capita Municipality Income				-0.00023*	-0.00012	-0.00007	-0.00018
				(0.00012)	(0.00017)	(0.00017)	(0.00025)
Constant	0.00726***	0.00695***	0.00672***	0.01057***	0.01236***	0.01281***	0.00953***
	(0.00013)	(0.00012)	(0.00012)	(0.00041)	(0.00059)	(0.00061)	(0.00080)
Age-Time FE	NO	YES	YES	YES	YES	YES	NO
Municipality-Time FE	NO	NO	YES	NO	NO	NO	NO
Municipality Controls	NO	NO	NO	NO	YES	YES	NO
Number of Cases Control	NO	NO	NO	NO	NO	YES	NO
Observations	20,766,260	20,766,260	20,766,260	20,755,375	20,755,375	20,745,695	3,372
Adjusted R-squared	0.00019	0.00060	0.00064	0.00061	0.00063	0.00063	0.10132

Robust standard errors in parentheses
*** $p<0.01$, ** $p<0.05$, * $p<0.1$

Notes: Notes are similar to Table 2. However, in this table, the sample includes individuals aged 40 to 64, and still excludes people living in collective households, or households with more than 10 individuals. Municipality controls now consist of: the fraction of 40-64 year olds living in single households, the fraction of 40-64 year olds that are Belgian-born, the density (inh/km2), and the fraction of 40-64 year olds older than 55, as well as the interactions of these with year 2020.

Accepted manuscript

ACCEPTED MANUSCRIPT

Figure A.2: Excess Mortality by Education and Country of Birth

A. Education, Aged 40-64 **B. Country of Birth, Aged 40-64**

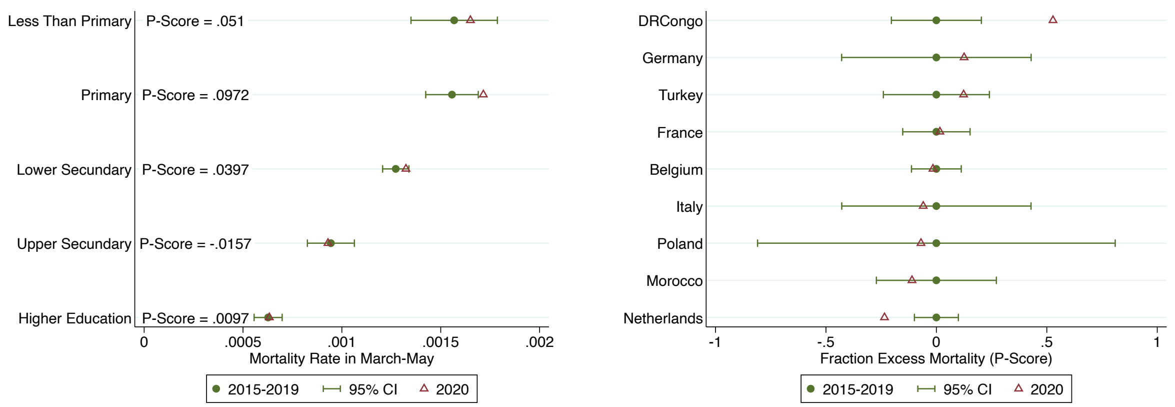

Notes: Panel A shows mortality rates (with 95% confidence intervals) in March-May 2015-2019 and March-May 2020 by educational level for individuals aged 40-64, excluding people living in collective households, or households with more than 10 individuals. Panel B shows excess mortality fractions in March-May 2020 and 95% confidence intervals for 2015-2019 by country of birth for individuals aged 40-64 and older, excluding people living in collective households, or households with more than 10 individuals. Calculation of mortality rates and P-score is similar as in Figure 4.

Figure A.3: Excess Mortality by Country of Birth and Industry, Residualized for Income

A. Country of Birth, Aged 65+ **B. Industry, Aged 40-64**

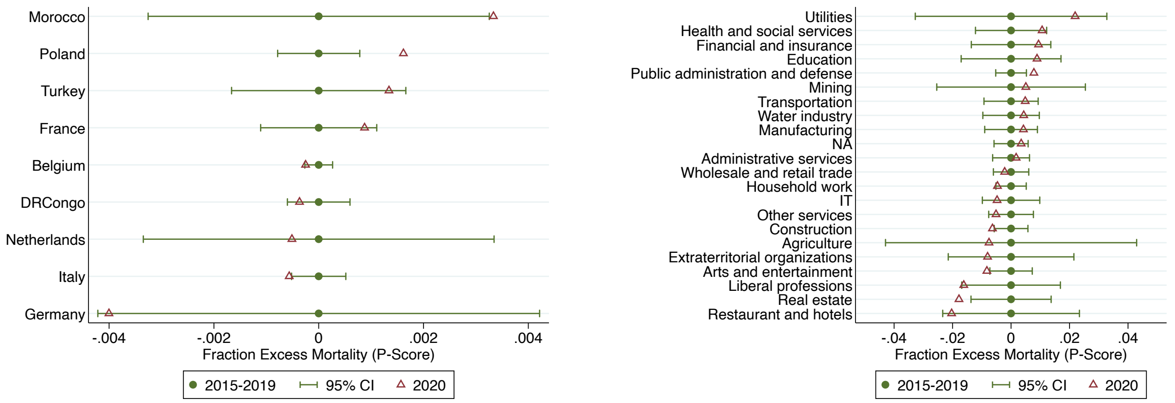

Notes: This Figure is similar to Panel B and Panel C of Figure 4, yet here the *residualized* (on income) mortality rates are plotted. Panel A shows these coefficients in March-May 2020 and 95% confidence intervals for 2015-2019 by country of birth for individuals aged 65 and older, after residualizing for household income decile. Panel B shows these coefficients in March-May 2020 and 95% confidence intervals for 2015-2019 by industry for individuals aged 40-64, after residualizing for household income decile. Samples in all panels exclude individuals living in collective households, or households with more than 10 individuals.
